# Molecular characterization and quantification using state of the art solid-state adiabatic TOBSY NMR in burn trauma

**DOI:** 10.3892/ijmm_00000288

**Published:** 2009-12-01

**Authors:** VALERIA RIGHI, OVIDIU ANDRONESI, DIONYSSIOS MINTZOPOULOS, A. ARIA TZIKA

**Affiliations:** 1 NMR Surgical Laboratory, Department of Surgery, Massachusetts General Hospital and Shriners Burn Institute, Harvard Medical School, Boston;; 2 Athinoula A. Martinos Center of Biomedical Imaging, Department of Radiology, Massachusetts General Hospital, Boston, MA 02114, USA

**Keywords:** nuclear magnetic resonance, high resolution magic angle spinning, TOtal through Bond correlation SpectroscopY, TOtal Correlation SpectroscopY, synchronized adiabatic inversion pulses, metabolite quantification, burn trauma

## Abstract

We describe a novel solid-state nuclear magnetic resonance (NMR) method that maximizes the advantages of high-resolution magic-angle-spinning (HRMAS), relative conventional liquid-state NMR approaches, when applied to intact biopsies of skeletal muscle specimens collected from burn trauma patients. This novel method, termed optimized adiabatic TOtal through Bond correlation SpectroscopY (TOBSY) solid-state NMR pulse sequence for two-dimensional (2D)^1^H-^1^H homonuclear scalar-coupling longitudinal isotropic mixing, was demonstrated to provide a 40–60% improvement in signal-to-noise ratio (SNR) relative to its liquid-state analogue TOCSY (TOtal Correlation SpectroscopY). Using 1-and 2-dimensional HRMAS NMR experiments, we identified several metabolites in burned tissues. Quantification of metabolites in burned tissues showed increased levels of lipid compounds, intracellular metabolites (e.g., taurine and phosphocreatine) and substantially decreased water-soluble metabolites (e.g., glutathione, carnosine, glucose, glutamine/glutamate and alanine). These findings demonstrate that HRMAS NMR Spectroscopy using TOBSY is a feasible technique that reveals new insights into the pathophysiology of burn trauma. Moreover, this method has applications that facilitate the development of novel therapeutic strategies.

## Introduction

Burn lesions are produced by a direct transfer of energy from any source of heat to body tissues. Severe thermal injuries are associated with marked metabolic alterations due to the liberation of inflammatory mediators and hormonal disturbances induced by stress. When a burn is sustained, there is a systemic release of adrenal stress hormones (catecholamines and glucocorticoids) to physiologically support the victim’s ability to fight and escape the threat (1). There is an increase in energy expenditure in burn injury attributable to metabolic processes, such as gluconeogenesis, ureagenesis, fatty acid (FA) synthesis, FA breakdown, Cory cycles, and processes working to compensate for loss of body heat through injured skin (2). Burn trauma that affects skeletal muscle has both local and systematic effects. Functionally debilitating changes were documented at local and distant sites; these changes are especially pronounced when the burn size exceeds 30% of the total body surface area (TBSA) (3).

NMR spectroscopy-based metabolomics analysis detects a wide range of metabolites in biological samples, enabling precise molecular screening (4–6). It was used extensively in studies of schizophrenia (6,7), Alzheimer’s disease (8), human brain tumors (9,10), and other human tumors (11,12). High Resolution Magic Angle Spinning (HRMAS) ^1^H magnetic resonance spectroscopy (^1^H-MRS) represents a promising, non-destructive tool for metabolic profiling of unprocessed tissue (13). NMR spectroscopy has been used to explore metabolic change after burn trauma in liver tissue extracts (14–16) and the HRMAS technique was recently used in combination with *in vivo* NMR to examine lipid accumulation following burn trauma (17).

Currently, HRMAS ^1^H-MRS of tissue biopsies employs conventional liquid-state pulse sequences. This approach assumes that MAS alone is sufficient to remove residual anisotropic interactions present in partially immobilized samples. This assumption holds true for simple one-dimensional (1D)^1^H-MRS. However, in multidimensional experiments that rely on ^1^H-^1^H homonuclear scalar-coupling (J-coupling) mediated magnetization transfer (i.e., TOtal Correlation Spectroscopy or TOCSY), residual anisotropic interactions are reintroduced unintentionally by pulse sequences. This degradation dramatically alters transfer efficiency, diminishing sensitivity, which is crucial in order for HRMAS ^1^H-MRS to become a routinely used diagnostic technique.

The diminished sensitivity problem is critical because multidimensional spectroscopy is necessary for unambiguous assignment and quantification of metabolites present in ‘crowded’ and overlapping 1D spectra. An optimized adiabatic TOtal through Bond correlation SpectroscopY (TOBSY) solid-state NMR pulse sequence for two-dimensional (2D)^1^H-^1^H homonuclear scalar-coupling mixing may yield a substantial signal-to-noise (SNR) gain relative to its liquid-state analogue TOCSY sequence (18). To this end, we developed and implemented the adiabatic 2D TOBSY solid-state NMR method in order to investigate burn metabolic injury. We compared 2D TOBSY to more conventional liquid-state NMR approaches and quantified the metabolites detected in burn trauma.

## Materials and methods

### Burn trauma mouse model

C57 mice were injured using an established burn trauma model (19,20). The experimental protocols were approved by the Massachusetts General Hospital Institutional Animal Research Review Board Committee. Mice were anesthetized by intraperitoneal injection of 40 mg/kg phenobarbital sodium. An area of the left leg corresponding to 5% of the total burn surface area (TBSA) was shaved and the burn injury was inflicted by immersing the left leg of mice in 90°C water for 4 sec. Three days after infliction of the burn, mice were sacrificed and the skeletal muscle tissue underlying the burn and contralateral muscle from the non-burned leg were harvested, immediately frozen in liquid nitrogen, and stored at −80°C.

### Ex vivo ^1^H HRMAS MRS

All HRMAS ^1^H MRS experiments were performed on a wide-bore Bruker Bio-Spin Avance NMR spectrometer (600.13 MHz) using a 4-mm triple resonance (^1^H, ^13^C, ^2^H) HRMAS probe (Bruker). The tissue samples (15–25 mg) were placed into a zirconium oxide (ZrO_2_) rotor tube (4 mm diameter, 50 *μ*l). A 10-*μ*l aliquot of external standard trimethylsilyl-propionic-2,2,3,3-d4 acid (TSP), (Mw=172, δ=0.00 ppm, 50 mM in D_2_O) that functioned as a reference for both resonance chemical shift and quantification was introduced into the rotor tube. The samples were secured and tightened in the rotors with a top insert, screw, and cap (Bruker). The HRMAS ^1^H MRS was performed at −8°C at a MAS speed of 3 kHz to minimize tissue degradation.

**Figure 1:**
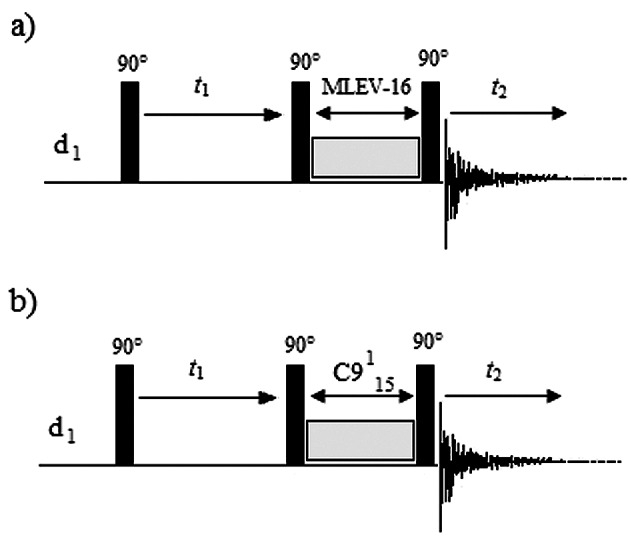
Pulse sequence for 2D [^1^H-^1^H] TOCSY (a) and TOBSY (b). In the TOSCY sequence, the core of the experiment is a spin-lock time (MLEV-16); this mixing time must be held long enough to complete the transfer process to all of the spins. The TOBSY experiment employs an adiabatic C9^1^_15_ rotor-synchronized pulse sequence. The mixing time for TOBSY and TOCSY are similar, on the order of 45 min.

The 1D water-suppression spin-echo Carr-Purcell-Meiboom-Gill (CPMG) pulse sequence [90°-(τ-180-τ)n-acquisition] was employed (21). Incorporation of the CPMG sequence is preferred over the use of simple Free Induction Decays (FIDs) because it acts as a T2 filter that reduces the interference of very broad features in the spectrum baseline originating from tissue water and macromolecules. The CPMG sequence protocol included an inter-pulse delay (τ=2π/ω_r_) of 333 *μ*sec, 256 transients, a spectral width of 7.2 kHz, 8k data points, and a 3 sec repetition time (TR). For quantification purposes, we measured the T2 relaxation time by varying the CPMG evolution time (T_CPMG_ = 2nτ) [n=7–800 (∼5–530 msec)]. We refined the C9^1^_15_ (TOBSY) symmetry-based ^13^C MAS solid-state NMR pulse sequence for 2D HRMAS ^1^H-MRS use, and compared the magnetization transfer efficiency and SNR to MLEV-16 (TOCSY) in burn mice specimens. C9^1^_15_ cancels the 1st order average Hamiltonian and minimizes the higher orders’ contributions from chemical shielding anisotropy (CSA), dipolar coupling (D) and offset terms, retaining only the isotropic J-coupling. In both cases, WURST-8 adiabatic inversion pulses were employed for their efficient use of radio-frequency (r.f.) power in the compensation of pulse offsets, in-homogeneity, and miscalibration with reduced r.f. heating. The C9^1^_15_ is a rotor-synchronized sequence with a fixed WURST-8 pulse length to rotor period ratio of 15/18, according to WURST-inverse-WURST (WiW) scheme (22). The 2D TOBSY pulse sequence using C9^1^_15_ is shown in Fig. 1 (a cw water suppression block was omitted) (18). For the 2D [^1^H,^1^H] TOCSY experiment, the C9^1^_15_ mixing block in Fig. 1 was replaced by the MLEV-16 scheme. Each 180° pulse of the MLEV-16 (23) was produced from a rotor synchronized WURST-8 adiabatic pulse, as described previously (24).

TOBSY and TOCSY sequences were acquired for the 2D [^1^H,^1^H] experiment. In all experiments, identical acquisition and processing parameters were used, 2k points along the direct dimension (13 ppm spectral width), 200 points along the indirect dimension (7.5 ppm spectral width), 8 scans, 2 dummy scans, 1-sec CW low-power on-resonance water pre-saturation, 2-sec total repetition time period, 45-min mixing time (to allow maximum buildup signal for most metabolites), 56-min total acquisition time; QSINE = 2 window function in both dimensions, FT with 2k points in the direct dimension and zero-filling to 1k in the second dimension, phase correction in both dimensions, and baseline correction in the second dimension. Spectra were acquired using XWINNMR 3.5 software (Bruker Biospin Corp, Billerica, MA).

### Ex vivo ^1^H HRMAS MRS data processing

MR spectra of specimens were analyzed using MestReC software (Mestrelab Research). A line-broadening apodization function of 0.5 Hz was applied to CPMG HRMAS ^1^H FIDs prior to Fourier transformation (FT). MR spectra were referenced with respect to TSP at δ 0.0 ppm (external standard), manually phased, and a Whittaker baseline estimator was applied to subtract the broad components of the baseline. The 1D slices of metabolites, extracted along the indirect dimension from 2D TOBSY and TOCSY, were scaled to a common noise level and the peaks were integrated using the XWINNMR software package (XWINNMR 3.5, Bruker Biospin Corp, Billerica, MA).

The 2D process parameters were, QSINE = 2 window function in both dimensions, FT with 2k points in the direct dimension and zero-filling to 1k in the second dimension, phase correction in both dimensions, and baseline correction in the second dimension. Spectra were processed using XWINNMR 3.5 software (Bruker). The 2D spectra were quantified using the Sparky program (T.D. Goddard and D.G. Kneller, SPARKY 3, (USCF, http://www.cgl.ucsf.edu/home/sparky).

### Quantification of metabolites

Metabolites were quantified using the ‘external standard’ technique in order to achieve more accurate values. We initially used 1D spectra for quantification. All peaks that appeared to be separate in the TOBSY spectrum but were not well resolved using the CPMG sequence were quantified using 2D TOBSY spectra (25).

### Absolute quantification of metabolites from 1D CPMG spectra

Resonance intensities were measured for -CH_3_ protons of the TSP and compared to the resonance intensities measured for metabolites. The peak intensities for most of the metabolites, as well as TSP, were calculated from the intensity of the respective resonance (X) measured from the T2-filtered HRMAS ^1^H MR spectrum. The calculated peak intensities were then corrected for T2 relaxation, using Ic(X)=Ir(X) x exp(T_CPMG_/T2(X))/n, where Ir(X) is the measured intensity, T_CPMG_ is the CPMG echo time, and n is the number of protons in the functional group and corresponds to the resonance of the metabolite. In accordance with the ‘external standard’ technique employed (26), metabolite concentrations were quantified relative to the absolute concentration (*μ*mol) of the respective metabolite [M]=Ic(M)/(IcTSP(M) x wt), where wt is the weight of the sample in grams.

### Relative quantification of metabolites from 2D TOBSY spectra

To quantify more metabolites, we used the ratio of the Cross Peak Volume of the Metabolites [CVP(M)] to the TSP Diagonal Peak Volume [DPV(TSP)], as described previously (27). This ratio was divided by the source biopsy specimen’s weight (wt) to yield normalized metabolite intensities, Ic(M)=(1/wt) x CPV(M)/DPV(TSP). Performance gains were calculated by dividing (C9^1^_15_) TOBSY SNR values by (MLEV-16) TOCSY SNR values (SNR_C9_/SNR_MLEV16_) for each metabolite within each sample; the calculated values were then averaged over all samples within each group.

### Statistics

The data are reported as means ± standard errors. Statistical analysis was done using the Student’s t-test. A p-value <0.05 was considered statistically significant in all cases.

## Results

**Figure 2:**
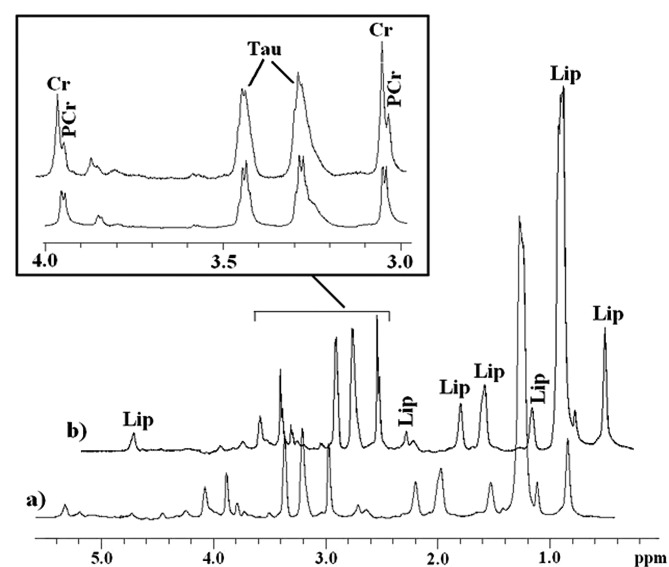
*Ex vivo* 1D HRMAS ^1^H CPMG spectra of (a) control and (b) burned skeletal muscle biopsies from mice. Lipid components: CH_3_ (0.89 ppm), (CH_2_)n (1.33 ppm), CH_2_C-CO (1.58ppm), CH_2_C═C (2.02 ppm), CH_2_C═O (2.24 ppm), ═CCH_2_C═ (2.78 ppm), CH═CH (5.33 ppm). The insert shows Creatine (Cr), PhosphoCreatine (PCr), and Taurine (Tau).

Fig. 2 presents 1D ^1^H HRMAS CPMG spectra from control and burned skeletal muscle biopsies collected 3 days after the burn trauma was inflicted. Consistent with prior reports (10,28,29), the 1D spectra revealed the presence of principal lipid components [CH_3_ (0.89 ppm), (CH_2_)n (1.33 ppm), CH_2_C-CO (1.58 ppm), CH_2_C═C (2.02 ppm), CH_2_C═O (2.24 ppm), ═CCH_2_C═ (2.78 ppm), CH═CH (5.33 ppm)] and certain metabolites [creatine plus phosphocreatine (Cr+PCr); taurine (Tau)].

**Figure 3:**
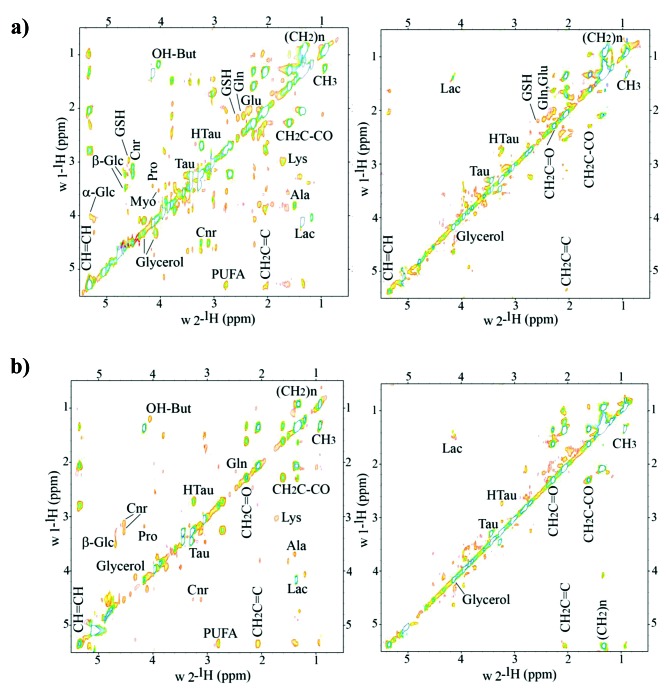
Representative 2D ^1^H-^1^H TOBSY (right) and TOSCY (left) HRMAS spectra of control (a) and burned (b) skeletal muscle. Several small metabolites and lipids were identified and assigned in both spectra. Lipid components, CH_3_ (0.89 ppm), (CH_2_)n (1.33 ppm), CH_2_C-CO (1.58ppm), CH_2_C═C (2.02 ppm), CH_2_C═O (2.24 ppm), ═CCH_2_C═ (2.78 ppm), CH═CH (5.33 ppm). Metabolites: alanine (Ala), lactate (Lac), OH-butyrate (OH-But), glutamine (Gln), glutamate (Glu), glutathione (GSH), Tau, hypotaurine (HTau), proline (Pro), lysine (Lys), myo-inositol (Myo), α-, ß-glucose (α-Glc, ß-Glc), carnosine (Cnr). We detected an altered concentration of several water-soluble metabolites in burned samples (b) relative to controls (a).

Metabolites that could not be assigned using the 1D spectra, were assigned using 2D TOBSY spectra (Fig. 3, right). Representative 2D NMR spectra of control and burned skeletal muscle specimens obtained with the (C9^1^_15_) TOBSY and (MLEV-16) TOCSY sequences are shown in Fig. 3a and b. Several small metabolites and lipids were identified and assigned in both types of spectra. We identified the following metabolites: alanine (Ala), lactate (Lac), OH-butyrate (OH-But), glutamine (Gln), glutamate (Glu), glutathione (GSH), Tau, hypotaurine (HTau), proline (Pro), lysine (Lys), myo-inositol (Myo), α-, ß-glucose (α-Glc, ß-Glc), carnosine (Cnr). Notably, several water-soluble metabolites were detected at altered concentrations in the burned samples (Fig. 3b) relative to the control samples (Fig. 3a). Some metabolites detected in control specimens, such as Glu, GSH and glucose, were absent or appeared only at trace levels in burned skeletal muscle specimens. The CPMG and TOBSY spectra were analyzed to determine chemical shifts and quantities of lipids, metabolites and osmolites that were characteristic of the burned tissues, and those calculated values are reported in Table I. Relative to control tissue, greater amounts of intramyocellular lipids (IMCLs, 1.33 ppm), ceramide (2.04 and 5.33 ppm) and polyunsaturated fatty acids (PUFA, 2.78 ppm) were observed in the burned tissue.

**Figure 4:**
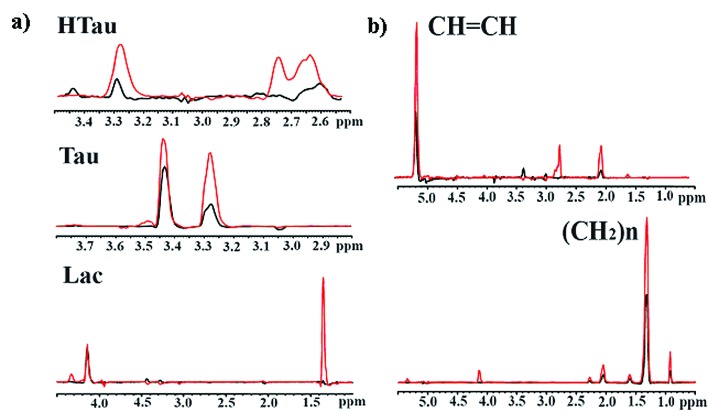
1D slices of selected metabolites were extracted along the indirect dimension from 2D experiment overlays of C9^1^_15_ (red) and MLEV-16 (black). (a) HTau, hypotaurine; Tau, taurine; Lac, lactate. (b) Unsaturated acids (CH═CH) and acyl chain methylene (CH_2_)n.

**Figure 5:**
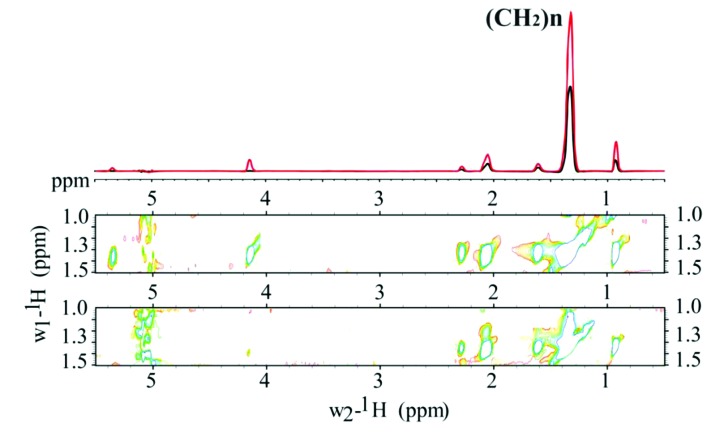
1D slices of acyl chain methylene (CH_2_)n and the relative spin system row for TOBSY (upper row) and TOCSY (lower panel).

As illustrated in Fig. 4, 1D slices were extracted along the indirect dimension of 2D TOBSY (red) and 2D TOCSY (black) experiments that analyzed control skeletal muscle specimens to confirm that the transfer efficiency predicted for the TOBSY sequence was met. 1D slices corresponding to both small metabolites (i.e., Lac, Tau, HTau) and large molecules [i.e., vinyl protons of FA chains (CH═CH) and intramyocellular lipids (CH_2_)n at 5.33 and 1.33 ppm, respectively] are shown in Fig. 4 (slices scaled to a common noise level and peaks integrated). C9^1^_15_ yielded higher signal intensities for low molecular weight metabolites (Fig. 4a), namely HTau and Tau (∼>50%), and Lac (∼>80%), as well as for high molecular weight metabolites (Fig. 4b), such as unsaturated acids (CH═CH) and intramyocellular lipids (CH_2_)n (∼>80%). An example of differential performance of the two 2D methods is presented in Fig. 5; note that the full FA (CH_2_)n spin system (six bonds), including the vinyl proton at 5.33 ppm (CH═CH), is apparent in the TOBSY spectrum but not the TOCSY spectrum.

**Figure 6:**
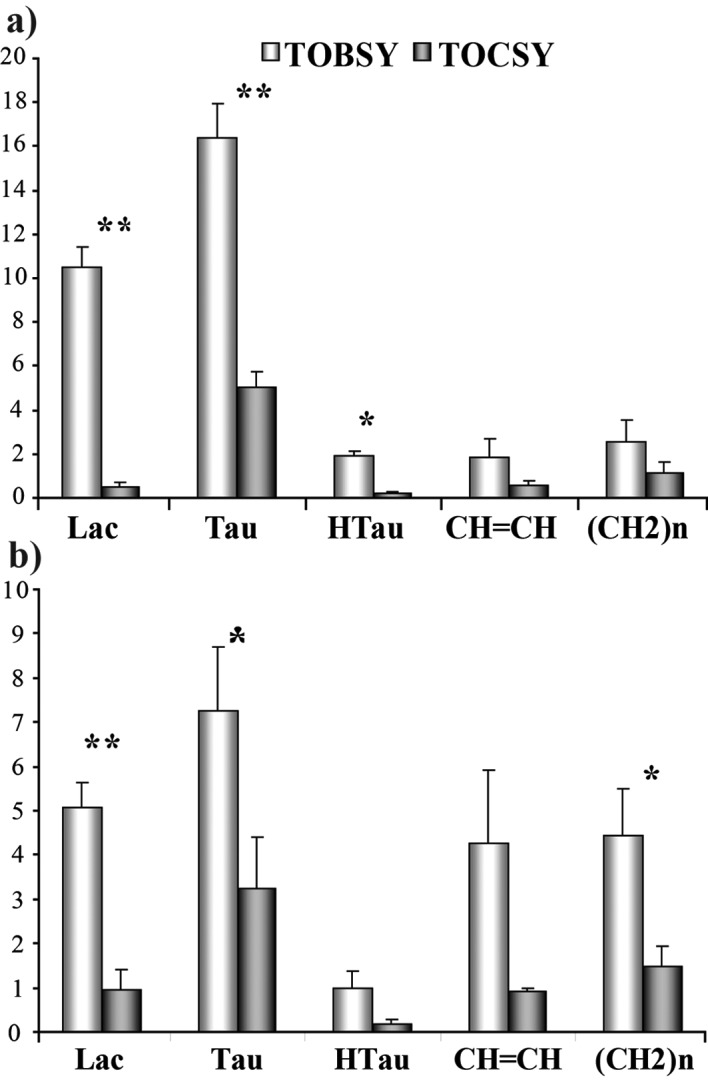
2D SNR gain of C91_15_ relative to TOBSY and TOCSY, for selected metabolites (Lac, lactate; Tau, taurine; HTau, hypotaurine; Lipids, CH═CH and (CH_2_)n) in a) control and b) burn skeletal muscle samples. ^*^p<0.05 and ^**^p<0.005 TOBSY vs. TOSCY, Student’s t-test. Y-axis, intensities in arbitrary units, x-axis, metabolites).

Averaging of the calculated 2D SNR ratio gains (SNR_C9_/SNR_MLEV16_) for each metabolite over multiple samples revealed that the use of (C9^1^_15_) TOBSY afforded a substantial SNR benefit over the use of (MLEV-16) TOCSY. The SNR gain for large molecules, such as FA components, was ∼60%, while that for low-molecular-weight, faster-tumbling meta-bolites (i.e., Tau, HTau and Lac) was slightly less, though still substantial, in the range of 40–50%. SNR gains for C9^1^_15_ relative to MLEV-16 in the 2D cross-peak volumes of selected metabolites in control and burned skeletal muscle samples are illustrated in Fig. 6.

## Discussion

In the present study, we demonstrate the utility of a novel 2D HRMAS NMR TOBSY method for gaining sensitivity in the detection of both small metabolites and lipids in burn trauma tissue. The novel (C9^1^_15_) TOBSY method decreased acquisition time and reduced metabolite concentration variability relative to (MLEV-16) TOCSY. Furthermore, it enabled us to detect new biomarkers of burn trauma biomarkers with 2D TOBSY.

### Performance of the TOBSY method

Use of the rotor-synchronized WURST-8 adiabatic pulse (C9^1^_15_) yielded operationally important improvements in SNR and resolution of tissue spectra relative to the isotropic mixing pulse (MLEV-16). Indeed, direct comparison between TOBSY(C9^1^_15_) and TOCSY(MLEV-16) spectra (Fig. 3) demonstrated enhanced sensitivity of the TOBSY NMR method to detect, identify, and quantify metabolites. The SNRs obtained with this new method were significantly better than those obtained using the conventional TOCSY sequence for both low- and high-molecular weight compounds in both control and burned skeletal muscle samples (Fig. 6). The improved metabolic profile of burned skeletal muscle achieved with 2D TOBSY indicates that this method is well suited to complement 1D CPMG in qualitative and quantitative analysis of metabolite concentrations in burned tissues as it will enhance evaluation of burn-associated metabolic dysfunction.

### Molecular changes associated with burn injury

The presently observed increase in mobile lipid molecules detected in the tissues 3 days after burn injury could be associated with cellular process such as inflammation, apoptosis and necrosis in agreement with a previous study (17). The elevated levels of IMCLs, in particular, in the burn tissue serve as a substrate for oxidative metabolism. This increase may be due to down-regulation of lipid oxidizing enzymes (29). Triglycerides (TGA), eicosanoid precursors, and important components of lipid bi-layer membranes were also detected in burned tissues (signal of bonded glycerol 4.10, 4.30 and 5.24 ppm). The presence of these molecules in the present experiment agrees with the 4.5-fold increases in TGA-FFA (free FAs) cycling that have been documented in burn patients. This increased TAG-FFA cycling is likely due to the lipolytic effects of circulating catecholamines as it, unlike glycolytic substrate cycling, is attenuated by ß-adrenoceptor blockade (30,31).

Burn injury is associated with enhanced systemic inflammatory reaction and oxygen radical production. The post-traumatic imbalance between oxygen species production and free radical scavengers determine the outcome of local and distant tissue damage and further organ failure (32). We observed the presence of several sulfur-containing amino acid antioxidants, such as GSH, Tau and HTau (33), which is expected during the anabolic phase in severely burned patients (34). The antioxidative activities of sulfur-containing compounds follow a general trend in that more highly reduced forms are stronger antioxidants and the number of sulfur atoms determine, at least in part, their modulatory activities on glutathione-related antioxidant enzymes (33). The increase of Tau in burned skeletal muscle is probably due to the capacity of Tau to help reduce levels of reactive oxygen species (ROS) and thus prevent changes in membrane permeability caused by oxidant injury. Recent studies report that Tau has protective effects on mitochondria and their enzyme activities in myocardium in rats that were subjected to a severe burn. These protective effects may be attributable to Tau’s ability to improve oxygen free radical eradication and alleviate Ca^2+^ overload in the mitochondria (35).

GSH, a cysteine-containing tripeptide synthesized from glutamate, cisteine and glycine, is a metabolite involved in oxidative stress. The significantly reduced GSH observed in the burned tissues relative to control tissues is likely attributable to general oxidative stress or oxidative damage. GSH is a major component of the cellular antioxidant system and plays an important role in the antioxidation of ROS and free radicals. Moreover, vulnerability to free radical damage was reported following GSH depletion in a number of cell systems (32).

The levels of HTau and Tau are probably determined by the redox balance; HTau is a precursor of Tau, the main product of Cys metabolism in mammals, and is thought to share the same physiological function (36). We speculate that HTau levels are reduced in burned tissues in favor of the production of Tau. Cnr is an endogenously synthesized dipeptide present in large amounts in skeletal muscle (37). It scavenges ROS as well as α-ß unsaturated aldehydes formed from peroxidation of cell membrane fatty acids during oxidative stress (15). Gln is an important mediator in numerous metabolic pathways and it acts as a regulator of some physiological processes including glycogen synthesis, gluconeogenesis and lypolysis. Gln plays a critical role as a signaling molecule in amino acid and glucose-stimulated insulin secretion. Dipeptide Ala-Gln promotes the turnover of glucose metabolism, signaling peripheral tissue to increase glucose utilization (38,39).

Our observation of increased PCr (Table I) in burn tissue provides support for the notion that PCr is increased in response to burn injury (40). Both the PCr and creatine increases that we observed are probably due to an elevated activity of creatine phosphokinase enzyme (CPK) which mediates the conversion of ATP to PCr and PCr’s breakdown to creatine and phosphorous. Indeed, CPK is elevated in burn victims, probably due to keratinocyte necrosis and leakage of CPK into circulation (40).

### Apoptosis

More research is required to resolve the relative importance and timing of apoptosis in muscle atrophy following a burn injury. Thermal injury was found to induce apoptosis in the skeletal muscle of rats as early as one day after burn injury (41). Correspondingly, Argiles and colleagues recently suggested that activation of apoptosis signaling is essential to and precedes protein degradation in skeletal muscle wasting during catabolic conditions (42). Skeletal muscle atrophy following burn injury was mostly due to a protein degradation mechanism principally involving the ubiquitin-proteosome pathway (43). It was also postulated that mitochondrial dysfunction and deregulation of apoptotic signaling plays a critical role in the development of sarcopenia of aging (44,45). The present observation of increased ceramide, a key apoptotic second messenger, in burn tissue presumably reflects burn-induced apoptosis. The presence of increased ceramide levels leads to the activation of stress-activated protein kinase, leading ultimately to activation of the pro-apoptotic factors caspase-1, -3, and -9 (41). Thus, growing evidence suggests that up-regulated expression and proteolysis is the result, rather than the cause, of burn-associated apoptosis.

In conclusion, we demonstrated that the presently introduced solid-state HRMAS TOBSY NMR method is a sensitive tool in the molecular characterization of metabolic perturbations in skeletal muscle after burn trauma. Increased FA levels detected in burn tissue reflects activation of inflammatory and apoptotic mechanisms that are directly relevant to mitochondrial dysfunction. Burn injury produced changes in metabolite levels that are attributed to oxidative stress. These findings provide insight into the pathophysiology of burn trauma studies and such findings can be used to direct research into novel therapeutic strategies.

## Figures and Tables

**Table I:** Chemical shift (δ, ppm) and quantity of selected metabolites in burned versus control skeletal muscle specimens.

Metabolite	δ^1^H (ppm)	Group	Control	Burn	% Δ from control^a^	P^b^
Lipid components	0.90	CH_3_	13.9±8.2^c^	36.9±4.7^c^	−90.6%	0.06
	1.29	(CH_2_)_n_	42.6±18.3^c^	155.7±43.9^c^	−114.1%	0.05
	1.58	CH_2_CCO	3.4±2.1^c^	7.5±1.4^c^	−75.2%	0.12
	2.03	CH_2_C═C	17.2±11.5^c^	155.4±28.6^c^	−160.3%	0.03
	2.24	CH_2_-CO	38.2±2.8^c^	77.2±18.2^c^	−67.6%	0.01
	2.78	═CCH_2_C═	3.0±1.9^c^	27.1±14.1^c^	−160.1%	
	5.33	CH═CH	4.1±3.8^c^	11.7±1.9^c^	−96.2%	0.01
OH-Butyrate	1.18	CH_3_	6.7±1.3^c^	4.7±0.7^c^	+35.9%	
Lactate	1.33	CH_3_	32.2±13.4^c^	59.5±4.5^c^	−60.0%	0.13
	4.11	CH				
Alanine	1.48	CH_3_	1.5±0.5^d^	0.6±0.3^d^	+85.7%	0.05
	3.78	CH				
Lysine	1.69	δCH_2_	2.8±0.4^d^	1.02±0.4^d^	+93.19	0.05
	3.01	ɛCH_2_				
Glutamate	2.09	ßCH_2_	0.6±0.1^d^	nd	-	
	2.35	γCH_2_				
Glutamine	2.17	ßCH_2_	1.1±0.3^d^	<0.14	-	
	2.44	γCH_2_				
Glutathione	2.55	γCH_2_-Glu	0.8±0.1^d^	nd	-	
	2.96	ßCH_2_-Cys				
	4.52	αCH_2_-Cys				
Hypotaurine	2.67	NCH_2_	3.8±0.6^c^	1.7±0.5^c^	+76.4%	0.12
	3.35	SCH_2_				
P-Creatine	3.02	CH_3_	5.3±2.2^c^	6.8±2.5^c^	−24.8%	0.09
Creatine	3.04	CH_3_	5.4±1.0^c^	10.4±7.5^c^	−63.3%	0.01
	3.93	CH_2_				
Carnosine	3.10	CH_2_-ring	2.0±0.4^d^	1.2±0.6^d^	+50.0%	0.02
	3.23	CH_2_-CO				
	4.50	CH-CO				
Taurine	3.26	S-CH_2_	14.1±2.2^c^	36.9±9.4^c^	−89.41%	0.15
	3.42	N-CH_2_				
Glycine	3.55	CH_2_	nd	nd	-	
Myo-inositol	4.08	2-CH	1.5±0.7^c^	nd		
	3.54	1,3-CH				
Proline	4.17	CH	1.7±0.8^d^	nd		
	3.43	CH				
ß-Glucose	4.67	1-CH	nd	nd	-	
	3.26	2-CH				
	3.48	4-CH				
α-Glucose	5.22	1-CH	nd	nd	-	
Glycerol	4.10	1-CH_2_	0.4±0.07^d^	1.4±0.6^d^	−111.1%	0.11
	4.30	3-CH_2_				
	5.24	2-CH				

a % Difference, tumor vs. control;

b Student’s t-test;

c concentration in *μ*mol/g from 1D CPMG;

d normalized ratio calculated from 2D TOBSY peak volumes (arbitrary unit); nd, non-detectable or traces.

## Abbreviations:

Ala: Alanine
Cnr: carnosine
CPMG: Carr-Purcell-Meiboom-Gill
Cr: creatine
CPK: creatine phosphokinase enzymes
FFA: free fatty acids
α-Glc, ß-Glc: α-, ß-Glucose
Glu: glutamate
Gln: glutamine
GSH: glutathione
HRMAS: high-resolution magic angle spinning
HTau: hypotaurine
Ile: isoleucine
Lac: lactate
Lip: lipids
Lys: lysine
Myo: myo-inositol
OH-But: OH-butyrate
PCr: phosphocreatine
Pro: proline
PUFA: polyunsaturated fatty acid
Tau: taurine
TOBSY: TOtal through Bond correlation SpectroscopY
TOCSY: TOtal Correlation SpectroscopY
TGA: triglycerides
